# Ni-Catalysts Supported on N,B-Doped Graphene Aerogels for CO_2_ Methanation

**DOI:** 10.1021/acsanm.5c03770

**Published:** 2025-11-06

**Authors:** M. Sánchez-González, A. Villardon, R. Campana, L. Sánchez-Silva, F. Dorado, A. Romero

**Affiliations:** † Department of Chemical Engineering, 16733 University of Castilla-La Mancha, Avda. Camilo José Cela 12, Ciudad Real 13071, Spain; ‡ Centro Nacional Del Hidrógeno, Prolongación Fernando El Santo, S/N, Puertollano, Ciudad Real 13500, Spain

**Keywords:** aerogels, heteroatom doping, graphene-based materials, Ni-catalysts, methanation

## Abstract

Methane synthesis from CO_2_ hydrogenation is a promising approach for CO_2_ recycling despite challenges such as nickel species loss and sintering. This study investigates reduced graphene aerogels (rGOA) doped with nitrogen (N-rGOA) and boron (B-rGOA) as supports for nickel-based CO_2_ methanation catalysts. Boron doping (Ni/B-rGOA) improved Ni dispersion and increased the number of active sites through structural and electronic modifications. However, it exhibited slightly lower catalytic performance than nitrogen doping (Ni/N-rGOA), which is attributed to larger Ni particles and higher surface acidity, hindering CO_2_ activation. ICP and XPS analyses revealed a higher Ni surface segregation in doped samples than in undoped Ni/rGOA. XPS also confirmed the presence of metallic Ni^0^ and Ni^2+^ species, with satellite peaks at 861 eV indicative of NiO. Boron doping modified the electronic structure of the carbon support, increasing Ni electron density and catalytic activity. TEM imaging showed well-dispersed Ni nanoparticles (5.9 to 7.3 nm) with no signs of aggregation. Among the tested catalysts, Ni/N-rGOA demonstrated superior CO_2_ conversion and CH_4_ selectivity, maintaining stable performance over 60 h of continuous operation. These findings underscore the potential of nitrogen-doped graphene aerogels as robust and efficient supports for the production of CO_2_ methanation catalysts.

## Graphical abstract

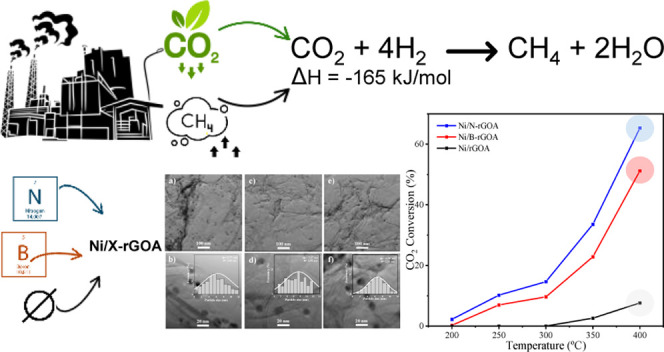

## 1 Introduction

The increasing levels of CO_2_ emissions represent a critical threat to the environment, contributing significantly to global warming and climate change.^1^ One promising solution is converting CO_2_ into methane, a process that not only helps reduce atmospheric CO_2_ but also generates a valuable renewable energy resource. By utilizing this method, we can mitigate the harmful effects of greenhouse gases while simultaneously producing a sustainable alternative fuel source.^2^

Power-to-methane technology converts CO_2_ into methane using renewable energy, offering multiple benefits. It drastically reduces greenhouse gas emissions, enables the storage of renewable energy in the form of methane for later use, and provides versatile fuel for heating, electricity generation, and transportation.^2^ Furthermore, this technology helps balance the energy supply and demand by storing excess renewable energy, enhancing grid stability. The necessity for new investments is significantly mitigated by the effective utilization of existing natural gas infrastructure, enabling its efficient deployment.^3^ Numerous studies have emphasized the importance of the CO_2_ methanation reaction within power-to-methane technology as a means of lowering CO_2_ emissions.^4^

Current research on CO_2_ methanation focuses on enhancing the efficiency and scalability of the process.^5^ Efforts have centered around developing advanced catalysts to improve reaction rates and selectivity, aiming for higher conversion efficiencies. Simultaneously, research seeks to optimize reactor designs and operating conditions to reduce costs and enhance the feasibility of large-scale applications.^6^ The overarching goal is to establish CO_2_ methanation as a viable solution for reducing greenhouse gas emissions and producing renewable methane on a commercial scale.

Compared with other renewable fuels, such as bioethanol and biogas, renewable methane is particularly promising. While bioethanol and biogas are considered low-carbon energy sources, their production processes still pose environmental challenges, including greenhouse gas emissions during fermentation and the need for biomass waste treatment.^7^ Methane, in contrast, offers a cleaner alternative. The CO_2_ hydrogenation process to methane, developed by Paul Sabatier in 1902, remains competitive for commercial applications due to its high energy efficiency and conversion rates. CO_2_ methanation is widely used in the coal, natural gas, ammonia, and hydrogen production industries, though its application in emerging energy sectors continues to develop.^8^

Metal-based catalysts, especially those using nickel (Ni),^9^ are central to CO_2_ methanation. Ni is preferred due to its efficiency and low cost compared to noble metals like ruthenium (Ru), although other transition metals such as iron (Fe) and cobalt (Co) have also been studied. While noble metals exhibit high activity and selectivity, their high cost has driven the development of more economical Ni-based catalysts. Ni catalysts demonstrate high activity at elevated temperatures; however, they are prone to deactivation due to particle agglomeration and coke formation.^10^ Therefore, developing improved Ni catalysts to prevent agglomeration and enhance metal dispersion is crucial for increasing process efficiency and catalyst longevity.^11^

The selection of catalyst support plays a pivotal role in CO_2_ methanation, as it significantly impacts the catalyst’s efficiency, selectivity, and stability. Carbon-based supports,^12,13^ particularly those derived from graphene, such as graphene aerogels, have attracted considerable attention due to their outstanding properties.^13^ Well-designed graphene aerogel supports can improve the dispersion of active metal sites, enhance the thermal conductivity, and create favorable conditions for CO_2_ adsorption and activation. Their high surface area and mechanical strength also contribute to increased resistance to sintering and catalyst poisoning, ultimately extending the operational lifespan. By optimizing the properties of graphene aerogels as supports, higher conversion rates and improved performance in CO_2_ methanation can be achieved.^7^

Additionally, doping graphene materials with heteroatoms such as nitrogen (N) or boron (B) can further enhance their effectiveness as catalyst supports in CO_2_ methanation.^14^ These dopants modify the electronic properties and surface chemistry of graphene, promoting stronger interactions between the support and active metal sites.^15^ For instance, nitrogen doping increases the basicity and electron density of the material, facilitating CO_2_ adsorption and activation. On the other hand, boron doping improves the thermal stability and catalytic activity by generating additional active sites.^16^ Overall, these modifications lead to higher catalytic efficiency, improved selectivity, and enhanced durability, making doped graphene aerogels highly promising for CO_2_ methanation processes.

In this work, three innovative Ni catalysts supported on graphene aerogels—doped with nitrogen (N), boron (B), or left undoped—were meticulously synthesized, each with a 10 wt % Ni loading. Through comprehensive characterization and catalytic performance testing in the CO_2_ methanation reaction, a detailed comparative analysis was conducted. This work focuses on the exploration of heteroatom (N or B) doping in reduced graphene oxide-based aerogels (rGOA), highlighting the critical role of doping in enhancing the catalytic efficiency of Ni-based carbon-supported catalysts and providing new insights into the mechanisms underlying their superior performance.

## 2 Materials and Methods

### 2.1 Materials

Graphite powder 99% purity (⌀ < 2 0 μm), sulfuric acid 96–98% purity (H_2_SO_4_), potassium permanganate (KMnO_4_), hydrochloric acid ≥3 7 wt % (HCl), boric acid 99.5% purity (H_3_BO_3_), and hydrazine monohydrate (NH_2_NH_2_·H_2_O) were supplied by Sigma-Aldrich. Hydrogen peroxide (33 wt %/v) (H_2_O_2_) and nickel­(II) nitrate 6-hydrate (Ni­(NO_3_)_2_·6H_2_O) were supplied by PanReac. Ethanol 96% v/v (CH_3_CH_2_OH) was supplied by VWR chemicals.

### 2.2 Synthesis of Graphene Oxide and Doped Graphene-Based Aerogels

Graphite was oxidized to graphene oxide (GO) using the Hummers method.^17^ A mixture of graphite and potassium permanganate (KMnO_4_) in a 3:1 ratio was stirred with 400 mL of sulfuric acid at 50 °C for 2–3 h. To stop the oxidation, 3 mL of H_2_O_2_ and 400 g of ice were added. The resulting mixture was filtered under a vacuum, washed with deionized water, ethanol, and HCl, and then dried at 60–70 °C.

For the synthesis of graphene-based aerogels (see Scheme 1), GO (0.8 g) was dispersed in water (400 mL) using sonication; after that, the GO solution was mixed with an adequate amount of a reducing agent, boric acid or hydrazine monohydrate, depending on the doping. After stirring, the mixture was subjected to a hydrothermal process in an autoclave at 180 °C for 12 h. The obtained hydrogels were freeze-dried and then calcined under a nitrogen flow at 600 °C for 1 h to achieve the final aerogel form.

**Scheme 1:**
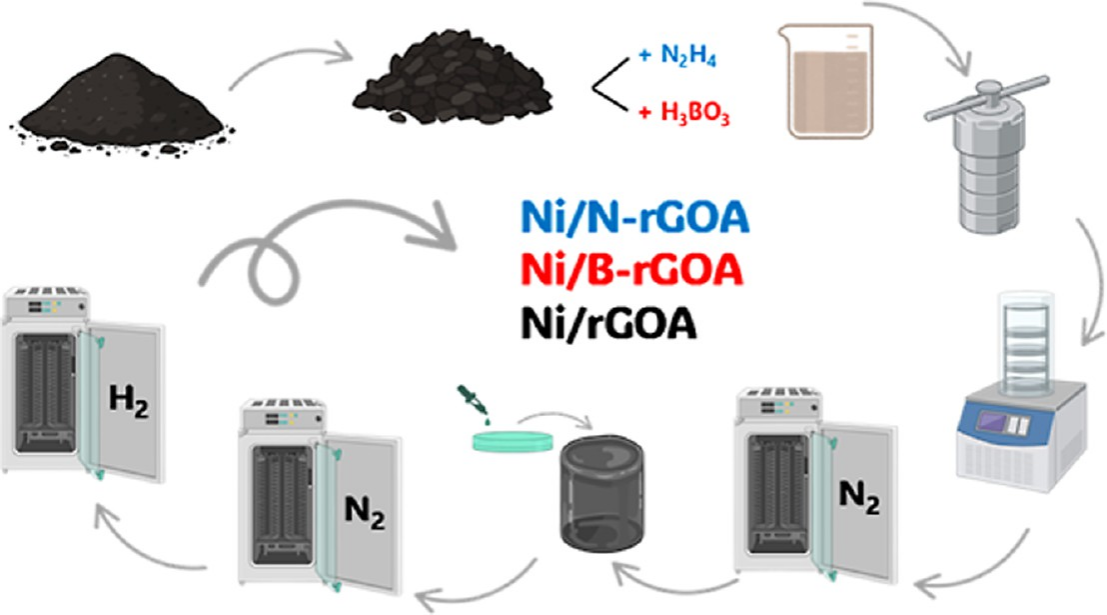
Schematic Process for Catalyst Synthesis

Additionally, a synthesis of the same graphene-based aerogels was performed without any doping (N/B) precursor for comparative purposes. This undoped aerogel followed the same preparation steps, allowing for a direct evaluation of the influence of doping on the material properties. The resulting samples were named rGOA, N-rGOA, and B-rGOA, with rGOA being the reduced graphene oxide aerogel, N-rGOA the nitrogen-doped reduced graphene oxide aerogel, and B-rGOA the boron-doped reduced graphene oxide aerogel.

### 2.3 Ni Catalyst Preparation

Nickel-supported catalysts were prepared by a wet impregnation method using nickel­(II) nitrate as the metallic salt with a Ni loading of 10%. First, a 1:1 solution of ethanol and deionized water (10 mL each) was prepared. The nickel nitrate needed to achieve a 10% nickel loading on the support was dissolved in this ethanol solution.^18^ The solution was then applied dropwise onto the support using a Pasteur pipet to ensure even coverage. The product was dried at 70 °C for approximately 20 min. This impregnation process was repeated until the solution was exhausted with careful mixing between applications to ensure uniformity. After the final impregnation, the support was left in an oven at 70 °C for 12 h. Finally, the Ni catalysts were calcined in a vertical furnace at 450 °C for 2 h (10 °C/min) and were denoted as Ni/N-rGOA, Ni/B-rGOA, and Ni/rGOA.

### 2.4 CO_2_ Methanation Reaction

The catalytic CO_2_ methanation tests were performed in a cylindrical stainless-steel fixed-bed tubular reactor (9.1 mm inner diameter) at atmospheric pressure, coupled to a Microactivity Reference Unit (PID Eng & Tech), which allows the control of reaction, reaction parameters, and data through mass flow controllers. Before each reaction, the catalyst was reduced to 400 °C for 60 min in H_2_. Reaction gases, including CO_2_ and H_2_ (with a H_2_/CO_2_ molar ratio of 4), were introduced into the reactor, with nitrogen (N_2_) as a carrier gas. Catalytic activity was tested under a gas hourly space velocity (GHSV) of 30,000 mL·g^–1^·h^–1^, over a temperature range of 200–400 °C, and reaction products were analyzed by gas chromatography with a thermal conductivity detector (GC-TCD). The turnover frequency (TOF) was determined by normalizing the reaction rate to the number of surface Ni atoms. Catalyst performance was evaluated in terms of CO_2_ conversion, CH_4_ selectivity, CH_4_ yield, and TOF using the following equations

1 $$X_{{CO}_{2}}\left(\%\right)=\left(\frac{{\left[{CO}_{2}\right]}_{0}-\left[{CO}_{2}\right]}{{{[CO}_{2}]}_{0}}\right)100$$

2 $$S_{{CH}_{4}}\left(\%\right)=\left(\frac{\left[{CH}_{4}\right]}{\left[{CH}_{4}\right]+\left[CO\right]}\right)100$$

3 $$yield\;\left(\%\right)={X_{{CO}_{2}}}^{*}S_{{CH}_{4}}$$

4 $$TOF\;\left(h^{-1}\right)=\left(\frac{F_{in}\cdot X_{{CO}_{2}}}{{Ni}_{s}}\right)100$$

where [CO_2_]_0_ denotes the initial CO_2_ concentration in the feed gas; [CH_4_], [CO], and [CO_2_] represent the methane, carbon monoxide, and carbon dioxide concentrations in the product gas, respectively. X_CO2_ corresponds to the CO_2_ conversion at each reaction temperature, F_in_ represents the feed gas flow rate, and Ni_s_ refers to the number of surface Ni atoms estimated from the average particle size determined by TEM. The turnover frequency (TOF) was calculated according to eq 4 by normalizing the reaction rate to the number of surface Ni atoms derived from TEM measurements, thus providing an intrinsic measure of catalytic activity independent of the total Ni loading.^19^

## 3 Supports and Catalysts Characterization

The Ni content in the final catalysts was analyzed through an inductively coupled plasma (ICP-OES, error of ±1%) spectroscopy (RL Liberty Sequential Varian equipment). Textural properties, such as surface area/porosity measurements, were analyzed via nitrogen adsorption/desorption at 77 K on a Quantachrome Quadrasorb SI system, with the pore size determined using the Barrett–Joyner–Halenda (BJH) method and surface area determined using the Brunauer–Emmett–Teller (BET) method at a relative pressure of *P*/*P*_0_ = 0.99. Prior to analysis, the samples were outgassed at 453 K in a gross vacuum for 6 h. Raman spectra were acquired from powdered samples using a 532 nm point-focused laser with the power density kept below 1 mW/μm^2^ to minimize laser-induced thermal effects. Measurements were performed using a Renishaw InVia Raman spectrometer. For each sample, spectra were collected at approximately 20–30 randomly selected points. To evaluate the degree of graphitization, the characteristic D and G bands of graphene were analyzed by fitting Lorentzian functions to the spectral data. X-ray diffraction (XRD) analysis was carried out using a PHILIPS PW-1711 diffractometer with CuK_a_ radiation (λ = 0.15404 nm), and crystallographic parameters such as in-plane crystallite size (*L*_A_) and crystal stack height (*L*_c_) were calculated through eqs 5 and 6. The Ni crystal size was calculated with the Scherrer equation based on fwhm’s of the Ni(111) diffraction peaks.

5 $$L_{C}=\frac{k_{C}\cdot \lambda }{{FWHM}_{1}\cdot \cos\theta_{1}}$$

6 $$L_{A}=\frac{k_{A}\cdot \lambda }{{FWHM}_{2}\cdot \cos\theta_{2}}$$

where fwhm is the full width at maximum height of the corresponding diffraction peak (rad); λ is the radiation wavelength (λ = 0.15404 nm); *k*_C_ is the factor (*k* = 0.9); *k*_A_ is the factor (*k* = 1.84); θ_1_ is the (0 0 2) diffraction peak position (°), and θ_2_ is the (1 0 0) diffraction peak position (°).

The reducibility of the samples was evaluated using hydrogen-temperature-programmed reduction (H_2_-TPR). The experiments were conducted using a Micromeritics AutoChem 2950 HP analyzer equipped with a thermal conductivity detector (TCD). Each calcined sample was placed in a U-shaped tube and outgassed by heating at 20 K/min under argon flow (50 mL/min) to 523 K. After cooling to room temperature, the samples were reduced with a 5 v/v % H_2_/Ar gas mixture (60 mL/min) at a heating rate of 10 K/min up to 1173 K, with peak values recorded by the TCD. CO_2_ temperature-programmed desorption (CO_2_-TPD) experiments were performed using a commercial Micromeritics AutoChem 2950 HP unit with a TCD to evaluate the basicity of the catalysts. A portion of the calcined sample was first reduced under a 5% v/v H_2_/Ar gas stream (60 mL·min^–1^) at a heating rate of 10 °C·min^–1^ up to 400 °C. The sample was then cooled to 50 °C under an Ar flow (20 mL·min^–1^) exposed to CO_2_ (40 mL·min^–1^) at 50 °C for 30 min. Afterward, CO_2_ was replaced with argon for 1 h. XPS analysis was performed using a Thermo Scientific Multilab 2000 spectrometer equipped with a 110 mm hemispherical sector analyzer and a dual-anode X-ray source (Al K-alpha and Mg K-alpha with photon energies of 1486.7 and 1253.6 eV, respectively). Each sample was mounted on a sample stub by using double-sided copper adhesive tape. The samples were then placed in the FEAL chamber to degas for 1 day. Survey spectra and high-resolution core level spectra were recorded using the Mg K-alpha X-ray source at 15 eV and a 400 W pass energy. The core-level spectra were deconvoluted and fitted by using the CASAXPS software package. No surface sputtering with Ar ions was performed, and all measurements were taken for as-received samples. The structure of the aerogel was analyzed in depth using HRSEM images performed by a ZEISS Gemini SEM 500 FE-SEM with a PIN-diode BSE detector. Thermal properties of the catalysts were analyzed using thermogravimetric analysis (TGA) on a TGA2, Mettler Toledo. The samples were heated in nitrogen from 25 to 1000 °C at a rate of 10 °C/min and a constant flow of 90 mL/min under an inert atmosphere (N_2_). The morphology of the catalysts was analyzed by using transmission electron microscopy (TEM) on a JEOL JEM-4000EX unit operating at an accelerating voltage of 400 kV. The samples were prepared by ultrasonic dispersion in acetone, followed by the evaporation of a drop of the resulting suspension onto a holey carbon-supported grid. The nickel particle size was determined from the TEM images, and the surface-area-weighted diameter (*d*_s_) was calculated using the equation

7 $${\mathrm{d̅}}_{s}=\frac{\sum in_{i}d_{i}^{3}}{\sum in_{i}d_{i}^{2}}$$

where *n*_*i*_ represents the number of particles with particle diameter (d_*i*_). Measurements were taken for over 800 particles, revealing that all of the catalysts exhibited a Gaussian particle size distribution.

## 4 Results and Discussion

### 4.1 Characterization of the Graphene Aerogel-Based Catalysts

Table 1 presents the crystallographic parameters obtained from X-ray diffraction (XRD) analysis. The values of *L*_A_ and *L*_C_ were slightly higher in the doped supports, indicating an increased degree of graphitization. This structural enhancement contributed to a notable reduction in both the surface area and pore volume in the N-rGOA and B-rGOA samples compared to those of the undoped rGOA. The observed increase in graphitization is attributed to the effect of heteroatom doping, which facilitates the reorganization of graphene layers and promotes more ordered growth along specific crystallographic directions.

**Table 1:** Physico-chemical Characterization of the Graphene-Based Aerogels and Catalysts

XPS elemental composition (%)										
SUPPORT^a^ CATALYST^b^	C	N or B	O	Ni	BE Ni 2p3/2 Ni^2+^ (eV)					
N-rGOA	90	2	8							
Ni/N-rGOA	89	1	8	2	853.4					
B-rGOA	80	5	15							
Ni/B-rGOA	85	1	12	2	853.2					
rGOA	87		13							
Ni/rGOA	91.7		8	0.3	854.4					
XRD parameters				textural properties			Ni content, particle size, and dispersion			
SUPPORT^a^	*L*_C_ (nm)	*L*_A_ (nm)	*S*_BET_ (m^2^ g^–1^)	*S*_micro_ (m^2^g^–1^)	*V*_pore_ ^c^ (cm^3^ g^–1^)	d_pore_ ^c^ (nm)	Ni content, ICP (wt %)	d_XRD_ ^d^ (nm)	d_TEM_ ^e^ (nm)	D_Ni_ (%)
CATALYST^b^								BR/AR		
N-rGOA	3.3	9.4	174	0	0.429	46.9				
Ni/N-rGOA	3,2	13,4	195	0	0.501	51.3	11.6	5.9/4.6	5.8	17.5
B-rGOA	3.8	6.3	260	30.1	0.728	59.9				
Ni/B-rGOA	4	13,4	200	22.9	0.430	43.1	10.2	7.3/3.2	7.2	14.2
rGOA	2.6	5.0	489	85.1	1.26	56.3				
Ni/rGOA	1.7	5.9	249	41.9	0.60	48.7	11.7	6.9/4.0	6.4	15.8

a Supports calcined at 600 ° C.

b Ni catalysts calcined at 450 °C and H_2_-reduced at 400 ° C.

c BJH total pore volume at *P*/*P*_0_ = 0.99 and average pore diameter.

d Ni particle size calculated from the Ni(111) plane using the Scherrer equation. BR: before reaction; AR: After reaction.

e Ni particle size calculated from TEM images. Estimated Ni dispersion (dispersion = 1.01/d_TEM_).

Complementary insights were obtained from Raman spectroscopy, which was employed to evaluate the defect density through the intensity ratio of the D (≈1350 cm^–1^) and G (≈1585 cm^–1^) bands (*I*_D_/*I*_G_). The results revealed an increase in the *I*_D_/*I*_G_ ratio following the reduction of GO, suggesting the formation of additional defects and vacancies. These structural imperfections—such as grain boundaries and edge sites—are known to enhance catalytic activity by generating reactive reduced carbon atoms.^20^

Raman analysis of the rGOA support for the Ni-based catalysts yielded D and G band intensities of 0.999 and 0.990, respectively, resulting in an *I*_D_/*I*_G_ ratio of 1.00. For the B–rGOA-supported catalyst, the D and G band intensities were 0.999 and 0.716, giving an *I*_D_/*I*_G_ ratio of 1.07. For the N–rGOA-supported catalyst, the measured D and G band intensities were 1.000 and 0.935, respectively, corresponding to an *I*_D_/*I*_G_ ratio of 1.40.

The higher ID/IG ratio observed in the nitrogen-doped sample indicates an increased degree of local structural disorder, which is often associated with the introduction of defects that are beneficial for catalytic activity. At the same time, XRD analysis confirms that long-range crystalline order is maintained or is even slightly enhanced in the doped samples. This combination of preserved long-range order and local defects creates a dual structural environment that is particularly advantageous for catalytic applications as it allows a balance between electrical conductivity and chemical reactivity.

On the other hand, the physical properties of the supports and nickel catalysts were analyzed using nitrogen adsorption–desorption isotherms. Table 1 provides a summary of the specific surface area (*S*_BET_), micropore area (*S*_micro_), pore volume (*V*_Pore_), and average pore size (*d*_Pore_) for both the catalysts and the supports. As shown in the N_2_ adsorption–desorption isotherms in Figure 1, both the parent graphene aerogels and the Ni catalysts exhibit type II isotherms with an H3 hysteresis loop, characterized by a mixed, crescent-like shape and inclined, inverted cone form.^21^ Typically, type II isotherms are associated with nonporous and macroporous materials (pores >50 nm), although their S-shape resembles IUPAC’s type IV isotherms.^22,23^

**Figure 1:**
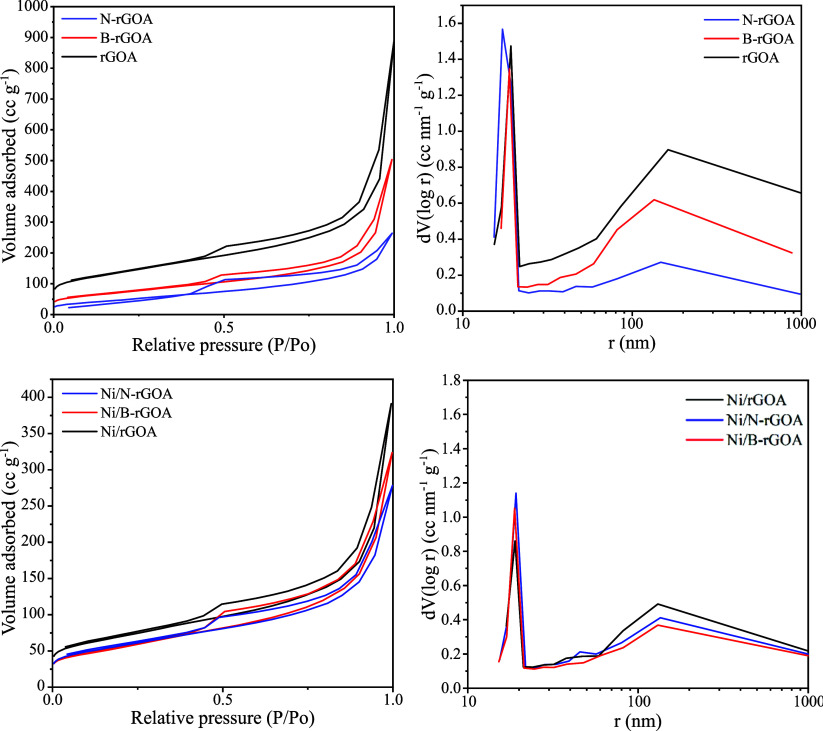
N_2_ adsorption–desorption isotherms and pore size distributions of graphene aerogel supports and Ni catalysts.

No significant change was observed in the shape of the N_2_ adsorption isotherm between the supports and catalysts, indicating that the original pore structure remained intact after Ni loading. However, a noticeable decrease in the amount of adsorbed N_2_ was seen after Ni loading, particularly in the Ni/rGOA sample and to a lesser extent in Ni/B-rGOA. This reduction is attributed to partial pore blockage by Ni particles, resulting in a lower surface area and pore volume. Interestingly, the Ni/N-rGOA sample exhibited slightly higher *S*_BET_ and *V*_Pore_ values compared to N-rGOA, suggesting that the Ni nanoparticles were more exposed on the surface of the support, providing additional geometrical surface area that contributes to the overall specific surface area of the final sample.^24^

The BJH pore size distribution, shown in Figure 1, confirms that all samples display a bimodal pore size distribution consisting of both macropores and mesopores. The macropores have significantly larger pore volumes, dominating the aerogel structure.^25^ These macropores range from 50 to over 1000 nm in radius, consistent with the typical 3D interconnected meso/macroporous structure seen in freeze-dried graphene aerogels, as further supported by SEM images (Figure 2). In contrast, the mesopores range between 10 and 20 nm in radius. The graphene aerogel-based supports and catalysts exhibit an average pore diameter of 45–60 nm (d_pore_, Table 1), confirming their meso/macroporous nature.

**Figure 2:**
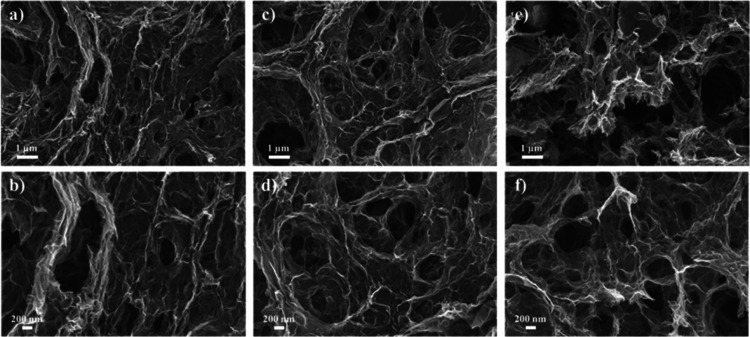
SEM images of graphene calcined aerogel-based supports: (a,b) N-rGOA, (c,d) B-rGOA, and (e,f) rGOA.

The metallic (nickel) content of the catalysts was determined by ICP-OES, and the corresponding data are collected in Table 1. As observed, the theoretical Ni content set at 10 wt % was slightly lower than the experimental nickel loading, which is probably related to some losses in support weight, associated with the reduction of its oxygen content during Ni introduction.^26^ This fact can be confirmed through the TGA of the calcined supports (Figure 3c), which shows a very small weight loss at temperatures near the subsequent calcination temperature of the Ni catalysts (450 ° C). By its part, TGA profiles of the Ni catalysts show a similar single degradation step, which is shifted to lower temperatures compared to the bare supports (Figure 3d). A comparison of Ni measurements from XPS (surface analysis, Table 1) and ICP (bulk analysis) indicates that Ni segregation to the surface was higher in the doped samples than in the undoped one. In other words, it can be concluded that in the Ni/rGOA catalyst, Ni was primarily located within the porous structure of the support, as only a very small amount of Ni was detectable on the surface by XPS.^27^

**Figure 3:**
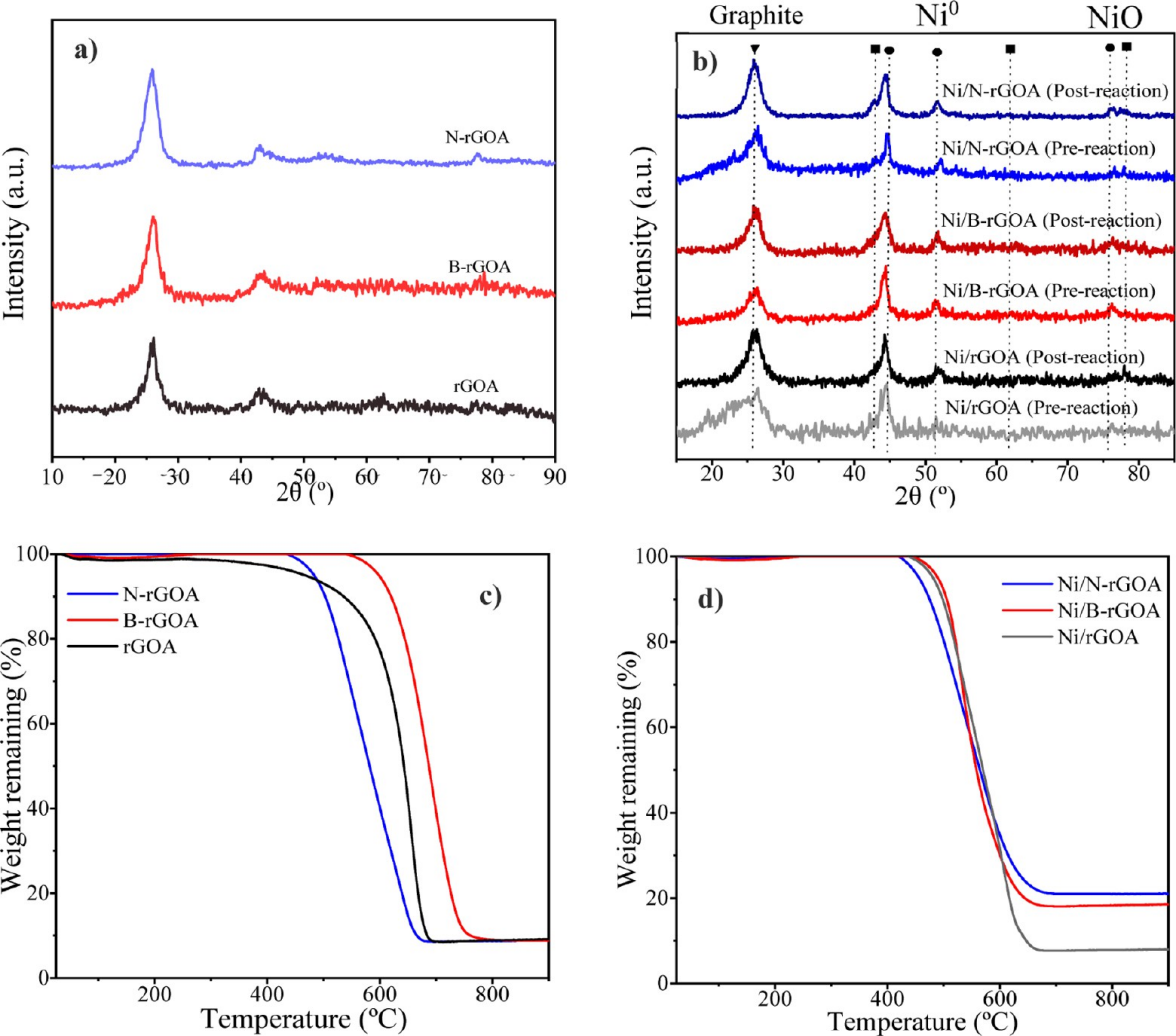
(a,b) XRD profiles and (c,d) TGA profiles of the graphene calcined aerogel supports and the reduced Ni catalysts.

Complementary XPS analysis (Table 1) obtained by Ni 2p_3/2_ spectra and its deconvolution was carried out (Figure 4). As it can be observed, the presence of a lower binding energy Ni 2p_3/2_ component between 852.3 and 853.5 eV can be assigned to metallic Ni^0^ species. Other components at around 856 eV are consistent with Ni^2+^ in NiO, along with satellite peaks from Ni^2+^ at 861 eV.^24^ It was demonstrated that, in the case of the nondoped sample, the overall signal intensity was much lower, and consequently, the noise was significantly more pronounced mainly in the satellite peak area, which was due to the low amount of surface Ni. On the other hand, all the peaks of the doped samples are shifted to lower binding energy values compared to the nondoped one (Table 1). As it has been previously described,^28,29^ a shift of the Ni 2p peaks to lower binding energy demonstrates an enhancement in the electronic density around Ni. Although boron is typically an electron acceptor, its doping in a carbon support can indirectly increase the electron density of nickel. This occurs by altering the electronic structure of the support, which enhances the metal–support interaction and facilitates electron transfer to Ni.^30^ The B 1s XPS spectra of the boron-doped graphene aerogel, shown in Figure 4e, confirm the presence of only one peak (192.75 eV) related to the substitution of a carbon atom by the B atom in the graphene layer. In this sense, the substitution of the C atom by the B atom, generating the BC_2_O bond, is related to the generation of structural defects in the carbonaceous matrix, and this could be associated with improvements in the activity of the final catalysts.^31^ Nevertheless, if nitrogen is used as a dopant, the effect on the electron density of nickel can differ due to nitrogen’s properties. Unlike boron, nitrogen is more electronegative and tends to act as an electron donor when incorporated into carbon-based materials, such as graphene supports. Nitrogen doping introduces functional groups (39% pyridinic, 50% pyrrolic, 8% quaternary, and 3% oxide of pyridinic, see Figure 4d) that can increase the electron density of the support, promoting electron transfer to Ni nanoparticles and improving its ability to adsorb and activate molecules like CO_2_, thus boosting catalytic activity.

**Figure 4:**
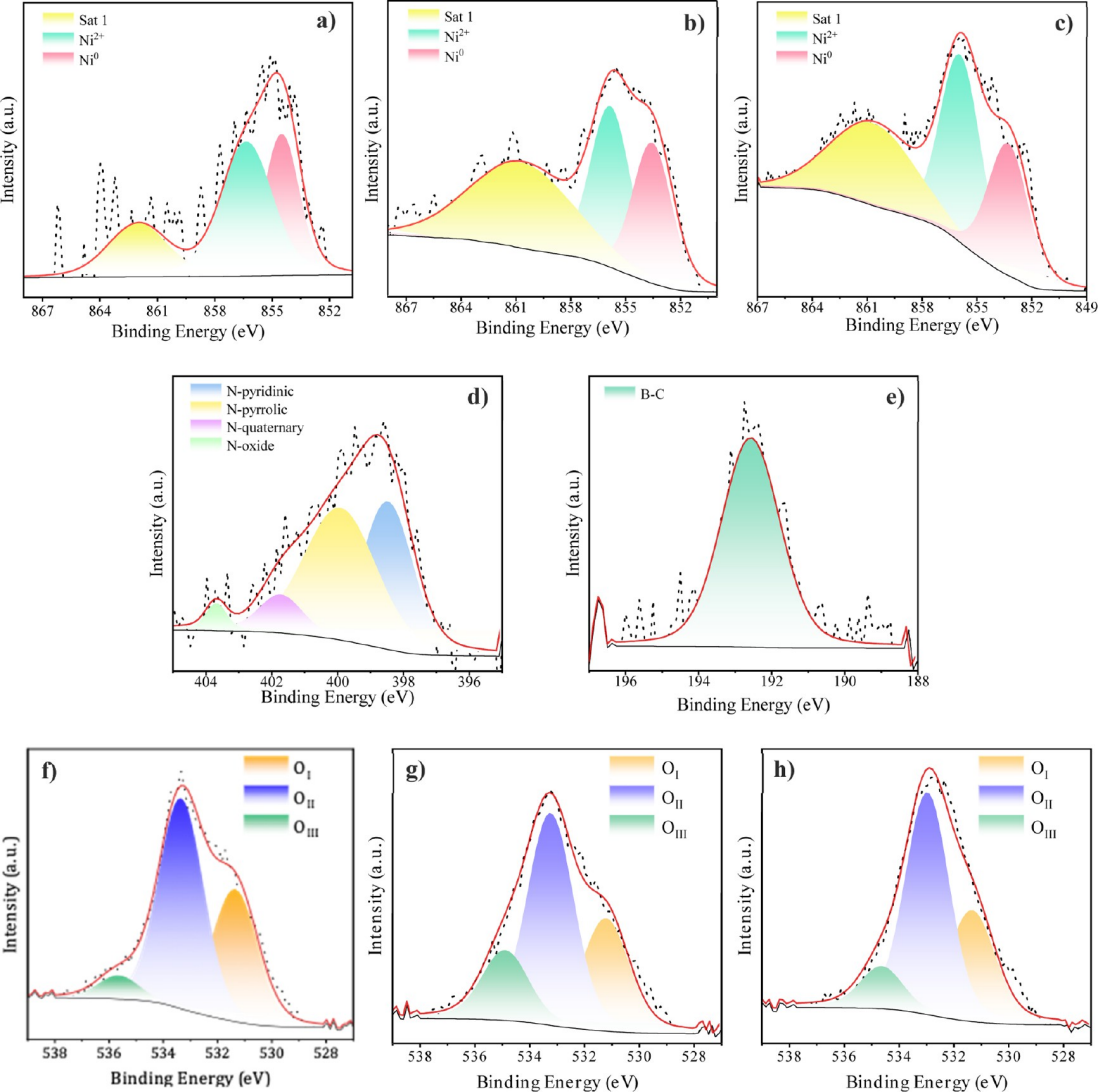
Ni 2p_3/2_ core-level spectra of reduced (a) Ni/r-GOA, (b) Ni/N-rGOA, and (c) Ni/B-rGOA; (d) N 1s core-level spectra of reduced Ni/N-rGOA; (e) B 1s core-level spectra of reduced Ni/B-rGOA; and O 1s spectrum of reduced (f) Ni/rGOA, (g) Ni/N-rGOA, and (h) Ni/B-rGOA.

It is important to note that the presence of an intense XPS peak corresponding to Ni^2+^ in the samples, despite the presence of Ni^0^, is attributed to the easy oxidation of Ni in the atmosphere during preparation and prior to introduction into the XPS measurement chamber. The identification of nickel oxide forms by XPS, along with the near absence of corresponding peaks in the diffraction pattern (below), can be explained by the poorly crystalline nature of the materials or the small particle size of NiO.^32,33^ The XPS spectra displayed in Figure 4f–h show the O 1s region, in which three main components can be distinguished: O_I_ (528.6 eV), assigned to lattice oxygen within the rGO structure exhibiting nucleophilic character; O_II_ (530.5 eV), attributed to surface hydroxyl (−OH) groups; and O_III_ (532.5 eV), corresponding to electrophilic oxygen species (O^2–^ or O^–^) associated with surface defects or adsorbed oxygen species.^34,35^ Notably, the N-doped sample exhibits a higher proportion of these electrophilic oxygen species, suggesting that nitrogen incorporation promotes the formation of surface defects. This observation is consistent with Raman analysis and may contribute to the enhanced catalytic performance observed.

Figure 5 presents transmission electron microscopy (TEM) images of three catalysts prepared by using different graphene-based aerogels as supports, along with their respective particle size distributions (inset). Independent of the graphene aerogel support used, the surface-averaged particle diameter (d_TEM_) was between 5.9 and 7.3 nm (Table 1), with the average Ni particle size being somewhat lower in the Ni/N-rGOA sample. The Ni-loaded catalyst’s structure demonstrates a thin sheet of the porous structure of the graphene aerogel with well-dispersed Ni nanoparticles on the surface.^21^ TEM images of the catalysts reveal no evidence of aggregated Ni particles on the surface,^36^ confirming the effectiveness of the preparation procedure used. Phase composition of the Ni catalysts was also investigated by means of XRD. Figure 3b shows that all catalysts exhibit a similar phase composition. In particular, the peak at 26° corresponds to the graphitic carbon of the graphene aerogels. Catalysts showed metallic Ni at 44.5° and 51.8° related to the (111) and (200) reflection planes, respectively.^20,37^ The absence or the low intensity of diffraction peaks of NiO at 37°, 43°, and 63°^38^ refers to the successful preparation of Ni^0^ on the support.^24^ Furthermore, the nickel deposition did not alter the position of the (002) reflection plane at 2θ = 26° (only a decrease in intensity was observed after Ni introduction, see Figure 3b), which implies that the nickel nanoparticles’ implantation does not influence the graphene aerogel crystal structure since the spacing between the layers stays unchanged.^39^ The Ni^0^ (111) crystallite sizes determined from the peak at 2θ = 43 ° were 5.9, 7.3, and 6.9 nm for Ni/N-rGOA, Ni/B-rGOA, and Ni/rGOA, respectively (d_XRD_, Table 1), roughly matching the TEM results.

**Figure 5:**
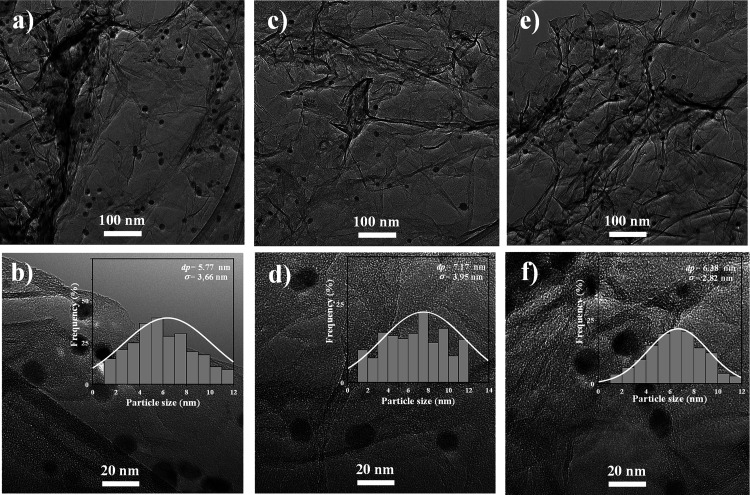
HRTEM images and particle size distribution of (a) and (b) Ni/N-rGOA, (c,d) Ni/B-rGOA, and (e,f) Ni/rGOA.

To analyze the Ni particle reduction conditions, H_2_-TPR analysis was carried out. The H_2_-TPR profiles of the aerogel-based supports are shown in Figure 6a. As indicated by the reducibility tests, a low H_2_ consumption was observed up to approximately 550 °C, followed by a significant increase. The gradual hydrogen consumption below 550 °C is attributed to the reduction of lattice oxygen on the carbon surface.^37^ In contrast, the increased H_2_ consumption at higher temperatures is associated with the degradation of the carbon matrix (e.g., carbon gasification).

**Figure 6:**
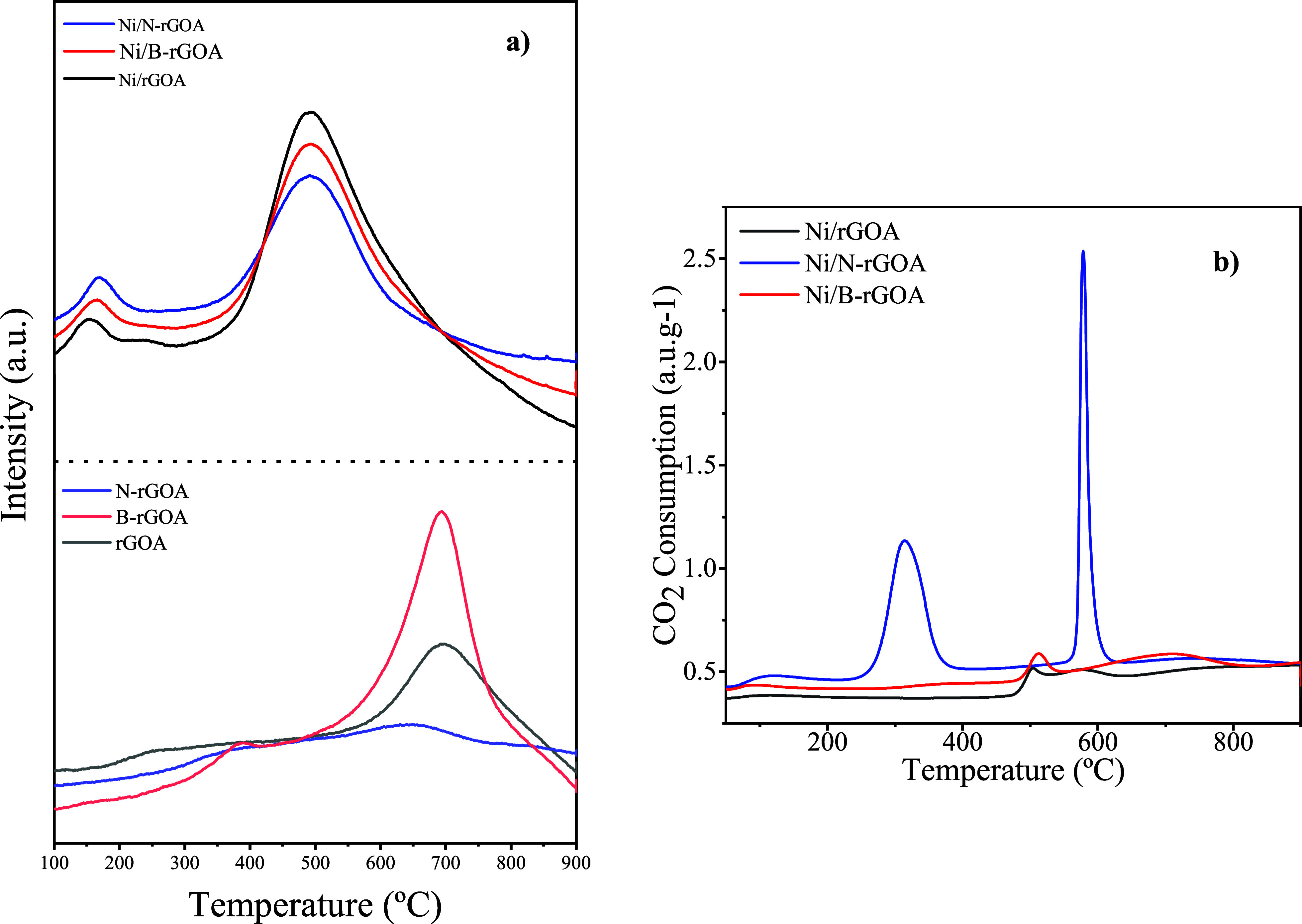
(a) H_2_-TPR profiles of graphene-based aerogel supports and Ni catalysts. (b) CO_2_-TPD profiles of Ni catalysts.

The TPR profiles of the Ni-catalysts revealed two distinct ranges of hydrogen consumption: one at low temperatures (100–300 °C) and another at higher temperatures (above 350 °C), regardless of the support used. The hydrogen consumption in the lower temperature range (100–300 °C) corresponds to the reduction surface amorphous NiO species^40^ and/or the reduction of NiO-promoted oxygen vacancies on graphene aerogels.^9^ In contrast, the strong signals observed up to 350° C could be attributed to the reduction of bulk NiO species with different extents of interactions with the support^41^ along with the gasification of the support. Generally, the reduction of bulk NiO (not reported) weakly making contact with the support occurs in the temperature range of 300–350 °C.^42^ Therefore, the shift to higher temperatures suggested the presence of Ni^2+^ species that strongly interact with the graphene support,^43,44^ which relates to the high dispersion of Ni on the reduced graphene aerogel support, also proven by EDX mapping (Figure 7). CO_2_-TPD analysis was conducted to evaluate the surface properties of the Ni-based catalysts supported on graphene aerogels, as shown in Figure 6b. The surface basicity,^41^ in terms of both the strength and density of basic sites, was determined from the temperature ranges and integrated areas of the desorption profiles.^42^ The desorption peaks were generally classified into three categories: weak, medium, and strong.

**Figure 7:**
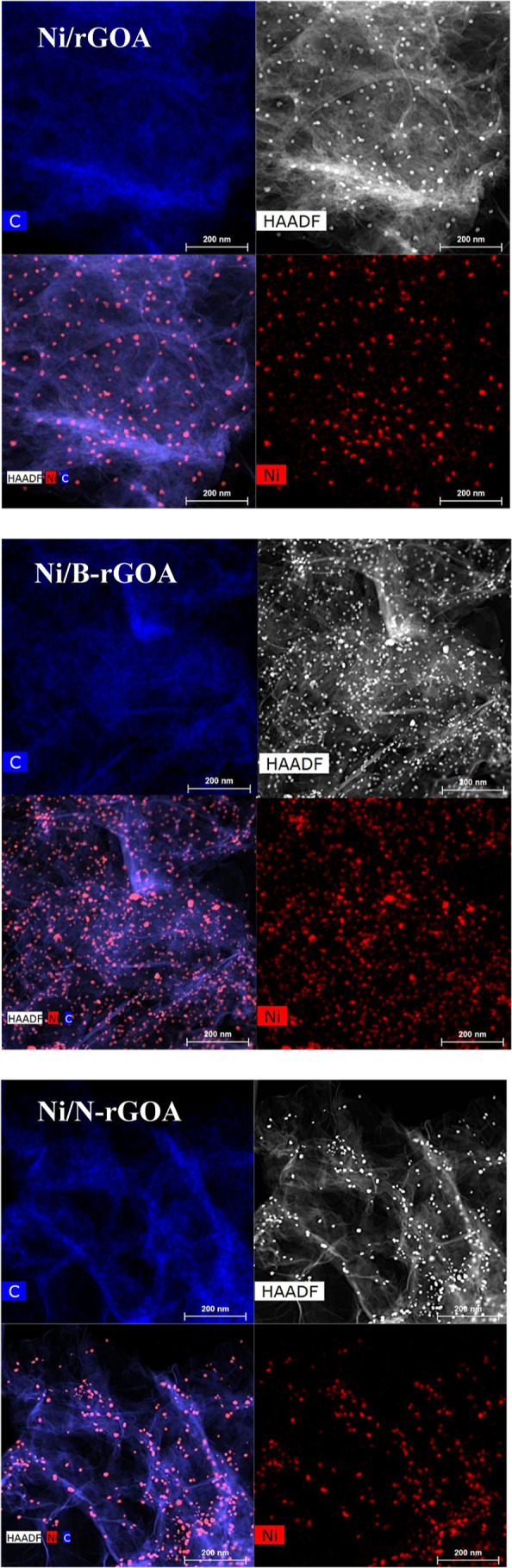
EDX elemental mapping of reduced Ni-based graphene aerogel catalysts.

For the Ni/N-rGOA catalyst, a desorption band appeared between 200 and 400 °C, indicating medium-strength basic sites, followed by a rather intense region around 600–700 °C, associated with moderately strong basic sites.^45^ The Ni/B-rGOA catalyst exhibited a weak-intensity region between 400 and 600 °C, corresponding to medium-strength basic sites, as well as a diffuse peak between 600 and 800 °C. For the Ni/rGOA catalyst, the region between 400 and 600 °C is also associated with medium-strength basic sites.

Since CO_2_ is mildly acidic, the presence of medium-strength basic sites facilitates its adsorption and activation. This feature is particularly advantageous in exothermic reactions such as CO_2_ methanation.^46,47^

It is also worth noting that the degradation of the carbon matrix in the bare graphene aerogels occurred at higher temperatures compared to that of the Ni catalysts. This indicates that the presence of nickel on carbon materials catalyzes the gasification of the carbon supports, likely due to the enhanced hydrogen activity on the surface of the Ni crystallites.^46^ The TPR data suggest that the reducibility of Ni is not significantly affected by the doping of the supports. These findings are consistent with the quite similar Ni particle sizes observed across the different support samples, as Ni reducibility is known to depend on Ni particle size.^48^

In this work, before the methanation tests, the catalysts were prereduced at 400 °C for 1 h under an H_2_ flow.^49^ This temperature was selected to prevent gasification of the support and based on the XRD results (Figure 3b), which confirmed that the NiO species in the catalysts were reduced to a high extent to metallic nickel at 400 °C.

### 4.2 Catalytic Tests

With well-characterized graphene aerogel-based catalysts, their catalytic properties in the CO_2_ methanation reaction were measured. CO_2_ conversion (X_CO2_) and selectivity (S_CH4_), yield to CH_4_, and turnover frequency (TOF) were investigated as a function of reaction temperature (Figure 8). Here, it is important to note that the catalytic results depend not only on the metal catalyst composition. The promoters/supports, the size of the metal particles, and the different experimental conditions applied (such as temperature, pressure, flow rate, catalyst mass, and reactor configuration) are some of the influencing factors.

**Figure 8:**
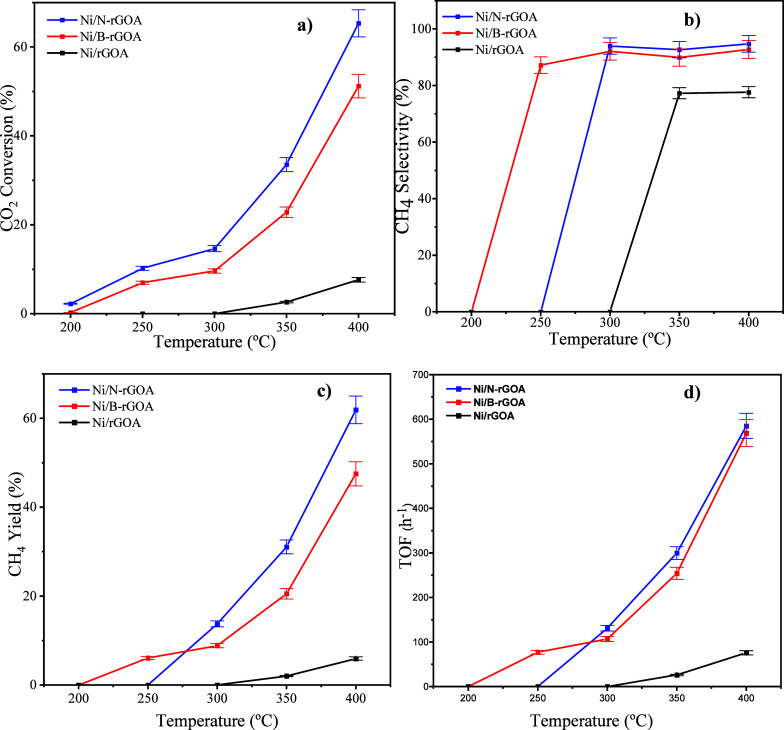
Catalytic evaluation of the catalysts: (a) CO_2_ conversion, (b) CH_4_ selectivity, (c) CH_4_ yield, and (d) TOF. Error bars represent the standard deviation (*n* = 3) (catalytic conditions: atmospheric pressure, 200–400 °C, GHSV = 30,000 mL·g^–1^·h^–1^, stoichiometric H_2_:CO_2_ ratio (v/v) = 4:1).

A direct comparison is challenging, as many catalysts reported in the literature are bimetallic systems or are doped with various promoters.^48,49^ Additionally, some studies were conducted under lower GHSV conditions, which typically result in higher CO_2_ conversion rates than those observed for the Ni-based rGOA catalysts presented in this work. Previous research has also demonstrated that heteroatom doping (e.g., B, N) can enhance the functional dispersion of transition metals by altering the surface chemistry of the support, even without a reduction in particle size.^50,51^

As can be seen, the presence of N or B as a doping agent of the catalytic support has a marked influence on the catalytic results. Micro-GC analysis revealed CH_4_ as the major product of CO_2_ methanation, with H_2_O formation confirmed by mass balance and only minor CO produced via the reverse water–gas shift reaction. No C_2_^+^ hydrocarbons were detected, and postreaction SEM images showed no significant carbon deposition, confirming the structural stability of the catalysts. These results highlight that the reaction proceeds predominantly via CO_2_ methanation, with high CH_4_ selectivity and negligible side products. As expected, the *X*_CO2_ starts to increase at a reaction temperature of 200 °C and continuously increases with the temperature for doped catalysts until the maximum operational temperature in this work (400 °C) is reached (Figure 8a). Interestingly, Ni/N–rGOA and Ni/B–rGOA catalysts achieved a higher *X*_CO2_ of 64% and 51% at 400 °C, respectively, when compared to Ni/rGOA that achieved a maximum *X*_CO2_ of 7% at 400 °C. Regarding methane selectivity, it was observed that when using the undoped graphene aerogel-based catalyst, selectivity values never exceeded 80%, even at 400 °C. However, when the catalytic support was doped with N or B, the results were completely different. The selectivity achieved by the N-doped catalyst was already 96% at 300 °C, whereas the B-doped catalyst did reach almost 90% CH_4_ selectivity at 250 °C (Figure 8b). Considering these results, the catalyst with the best performance for methane production was the one doped with N (Figure 8c).

Figure 8d shows the TOF values calculated from the reaction rates and Ni particle sizes determined by TEM. The TOF increases with the temperature for all catalysts, reflecting the enhanced participation of active sites at higher temperatures.

The results for the N-doped and B-doped catalysts are very similar, with a slight advantage for Ni/N-rGOA. This small improvement can be attributed to the additional basic sites introduced by nitrogen functionalities, which promote CO_2_ adsorption and activation. These results are consistent with the slightly higher CH_4_ selectivity observed for Ni/N-rGOA, confirming that the presence of nitrogen dopants not only enhances CO_2_ uptake but also enables more effective utilization of Ni active sites.^52^

After the catalytic tests, the catalysts were analyzed by using XRD (Figure 3b). The postreaction X-ray diffractograms revealed that the carbonaceous support became slightly more crystalline, as evidenced by a sharper and narrower (002) peak compared to the prereaction samples, along with higher values of crystalline parameters such as *L*_A_. Additionally, the Ni^0^ peak also appeared more defined yet slightly broader, suggesting a possible reduction in Ni particle size (noting that XRD measurements of Ni^0^ particle size are prone to significant errors due to small particle dimensions and signal noise). This behavior may be linked to the loss of oxygen that facilitates strong metal anchoring to the support, leading to the division of particles into smaller aggregates.^26^ During methanation, chemical reactants or intermediates (e.g., H_2_ or carbonaceous species) could further promote the redistribution of Ni particles.^52^ On the other hand, SEM images of the catalysts postreaction (Figure 9) showed surface morphologies similar to the bare supports (Figure 2), indicating minimal carbon deposition on the catalyst surface.

**Figure 9:**
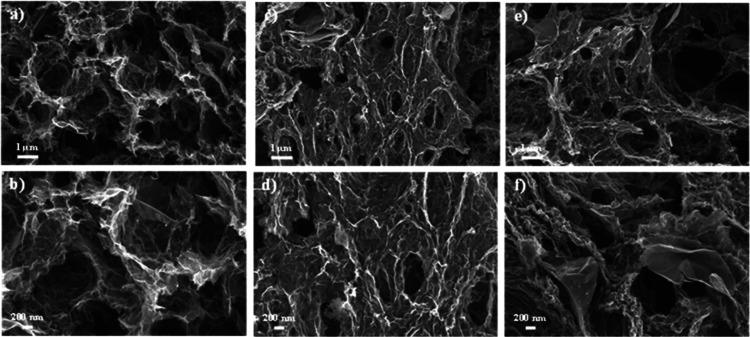
SEM images of the Ni-based graphene aerogels postreaction: (a,b) Ni/N-rGOA, (c,d) Ni/B-rGOA, and (e,f) Ni/rGOA.

For the short-term stability test, Figure 10 shows the 60 h stability test of the CO_2_ conversion and methane selectivity of the Ni/N-rGOA catalyst as a function of time at a reaction temperature of 400 °C and atmospheric pressure. The results show that the CO_2_ conversion is still around the initial result of 64% and that the methane selectivity remains around 96% for 60 h. It can be deduced from these experimental results that the catalyst has good stability for CO_2_ conversion and methane selectivity under the operating conditions adopted in this study. Additionally, everything seems to indicate that the changes observed by XRD after reaction do not affect the catalyst stability during the studied time frame.

**Figure 10:**
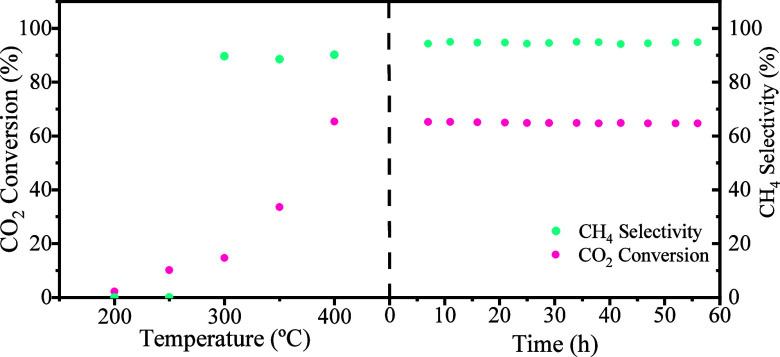
Catalytic stability of the catalyst Ni/N-rGOA.

Thus, this work demonstrates that N or B–doping of the rGOA aerogels leads to the development of a Ni-based catalyst with significantly improved performance toward CO_2_ methanation as compared to the nondoped ones. Additionally, the catalytic properties of the doped materials are comparable to the results reported in the literature, making graphene-based materials doped with heteroatoms such as N or B suitable for direct comparison with their undoped counterparts. This aims to elucidate the outstanding properties provided by N or B doping in graphene aerogels for the catalytic performance in CO_2_ methanation.

In this sense, it has been reported^38,53,54^ that the N sites in carbon supports could provide binding sites for the Ni species, enabling a good dispersion of the Ni precursor during impregnation and preventing agglomeration of the nanoparticles during the thermal treatment. Thus, the experimentally higher CO_2_ methanation performance of Ni/N-rGOA than that of Ni/rGOA observed in this work is a result of the lower size, higher dispersion, and accessibility of the catalytically active Ni phase over N–doped rGOA. This conclusion is supported by the multiple characterization methods that evidenced the existence of fine surface Ni catalytic particles well dispersed over N-doped catalysts (Table 1). In addition, the increased number of basic sites in the N-doped sample, primarily due to the presence of pyridinic-N (Figure 4d), is related to the improvement of the catalytic results.^55^

By its part, different from the most widely investigated nonmetallic N dopant, boron is less electronegative and is an important dopant that can exhibit novel properties. Therefore, boron-doped carbon supports have attracted widespread attention in many catalytic fields. Boron doping of carbon-based supports can significantly affect the catalytic activity of Ni-based catalysts in CO_2_ methanation. Boron introduces structural defects and electronic modifications to the carbon support,^56,57^ which can make the Ni active sites more accessible,^58^ increase the number of active sites, and modify the metal–support interactions. These factors may contribute to greater adsorption and activation of CO_2_ and hydrogen molecules on the catalyst surface, thereby improving both catalytic activity and selectivity toward methane production.^59^ Additionally, boron doping also increases the thermal stability of the support (compared to the undoped support), as corroborated by TGA results (Figure 3b), which is beneficial for high-temperature reactions such as methanation. As observed from the results, boron doping was not as catalytically effective as nitrogen doping (lower CH_4_ yield), which may be attributed to the slightly larger size of the Ni particles deposited on the support, which reduces the active surface area available for catalysis, leading to lower catalytic activity. Moreover, boron-doped carbon surfaces, being more acidic than those doped with nitrogen, could be less effective at activating CO_2_ for subsequent hydrogenation to methane.^56^

Although other strategies have been reported to obtain efficient catalysts—such as that described by Hu et al. (Ni/GA), which achieved ≈80% CO_2_ conversion and ≈95% CH_4_ selectivity at 350 °C—our results demonstrate that heteroatom-doped rGOA can deliver superior selectivity at lower temperatures while maintaining excellent stability, thus providing a valuable and complementary contribution to the development of advanced Ni-based methanation catalysts.^57^

## 5 Conclusions

This study demonstrates the significant impact of nitrogen (N) and boron (B) doping on the catalytic performance of Ni-based graphene aerogel (rGOA) catalysts for CO_2_ methanation. Compared to the undoped catalyst, N-doped rGOA (Ni/N-rGOA) exhibited superior catalytic activity, attributed to the better dispersion, reduced size, and increased accessibility of the Ni nanoparticles on the N-doped support. Furthermore, the presence of basic sites, particularly pyridinic-N, enhances the adsorption and activation of CO_2_, further improving catalytic performance. By its part, B doping (Ni/B-rGOA) introduces structural and electronic modifications that also improve Ni dispersion and increase the number of active sites. However, the boron-doped catalyst exhibited slightly lower catalytic yield compared to the nitrogen-doped counterpart, likely due to larger Ni particle sizes and higher surface acidity, which can limit CO_2_ activation. The postreaction analysis indicated that structural changes, such as enhanced crystallinity of the carbon support and smaller Ni particles, did not compromise catalyst stability. The Ni/N-rGOA catalyst maintained excellent CO_2_ conversion and CH_4_ selectivity over 60 h of operation, confirming its robustness under the studied reaction conditions. Overall, this work highlights the potential of heteroatom-doped graphene aerogels, particularly nitrogen-doped systems, as effective and stable supports for CO_2_ methanation catalysts. The insights into N and B doping effects provide valuable guidance for designing advanced catalysts for CO_2_ utilization in thermal processes to obtain renewable methane.
